# The Radcliffe Infirmary, Oxford

**Published:** 1891-04-18

**Authors:** 


### THE RADCLIFFE INFIRMARY, OXFORD.

We have received the following letter from the members of the Medical Staff of the Radcliffe Infirmary, Oxford, which we have much pleasure in publishing:—

The members of the staff of the Radcliffe Infirmary strongly protest against the unjust report upon the condition of the Infirmary published in your last issue. Your Commissioner who visited the Infirmary seems to have made a very casual and superficial inspection of it, or he could scarcely have made a report containing so many incorrect statements. We are informed, for he did not condescend to seek information from any of the members of the medical staff, that his visit was of about one hour's duration, and of this time one-half was occupied, by permission of the Matron, in giving an address to some of the nurses upon the advantage of their joining the "National Pension Fund for Nurses"; this would leave him a very short time for inspecting the wards, which are distributed rather widely over a large space of ground. We understand, moreover, that he only had conversation in the wards with one of the sisters, and did not inspect any of the lavatories or ward offices, or make any inquiries or examination as to the system of drainage, &c.

The front building of the Infirmary, plain but substantial, is undoubtedly old and somewhat out of date, but it only contains three of the wards with 15 beds in each. These wards, although old, are in no respect to be condemned as utterly bad wards; they are cheerful and commodious (1,309 cubic feet per bed), well lighted and ventilated, and by means of towers, erected some years ago, at their corners, have modern sanitary arrangements in the way of W.C.'s, sculleries, bath-rooms (these by the way contain Rufford's baths, as your Commissioner will be glad to learn) and lavatories. The rest of the beds are placed in separate ward buildings. The accident ward, built some twenty years ago, is a large ward containing 20 beds, and is supplied with ward offices such as were then considered to be of the best description,—the Parian cement in the walls was discoloured through faulty mixing of the colours, and it has so remained. A short time ago two squares of the ceiling, also Parian cement, fell down through their own weight, and were replaced by plaster with a coating of paint. If the Parian cement (used for its then supposed imperviousness) be impregnated through half its thickness, as your Commissioner suggests, with impurities of various kinds, they must be of a very innocent nature, for we get no evidence, in using the ward very largely, of any injurious effects upon the patients, such diseases as erysipelas and septicæmia being almost unknown amongst them. The Jubilee block connected to the front building by a corridor, which is warmed so as to be available for use by the patients in cold weather, contains two wards of 21 beds each. These are very excellent wards. The Parian cement upon their walls became discoloured, and has been colour-washed, to admit of its being frequently renewed, for we prefer this to paint, of which your Commissioner seems to be so fond. These wards are fitted with quite modern appliances in lavatories, bath-rooms (Rufford's baths), and sculleries; and we consider these so good, that we challenge your Commissioner to find better ones in any county hospital. The Leopold block containing a children's ward of 17 beds, is of modern construction, and has recently been extended; its sanitary arrangements are of modern fashion. This ward seems to have extracted a little faint praise even from your Commissioner. The fever block, an entirely separate building, is placed at the bottom of the garden; it contains 16 good beds, and has quite modern appliances (Rufford's baths), &c. The drainage of the whole series of buildings was renewed a few years ago under the direction of Messrs. Best and Co., sanitary engineers, at a cost of about £900, with the most approved arrangement of drains, syphons, and ventilated disconnecting chambers; and if your Commissioner will favour us with another visit, we will undertake to show him a system of drainage which will hold its own in comparison with that of any hospital in the kingdom. The system of nursing is one of entirely modern arrangement, the staff consisting of Matron, ward sisters, night sister, nurses, probationers, and ward-maids. The only Sister of whom your Commissioner deigned to speak in terms of praise is, curiously enough, the only one of the nursing staff who had seen fit to join the Pension Fund which he came to recommend. With regard to visitors in the wards, besides patients' friends, a visiting lady is appointed for each ward, and our complaint has been that a short time ago the amount of visiting was so great as to interfere with the work of the wards, and it was therefore somewhat curtailed. The restriction of expenditure by the Committee has not prevented the following alterations and additions to the hospital within something over twenty years:—A new accident ward and out-patient department, lavatory and scullery, towers to the wards in old building, new block of wards (Jubilee), Leopold block, which is an extension of children's wards, kindly given by a munificent Governor; fever block, new laundry, corridor connecting outlying wards with old building, a new system of drainage, adaptation of old wards for uses of nurses and probationers, and at the present time a scheme is under consideration for building another block in the garden to remove patients from wards in the old building, and give better accommodation for nurses. These, in addition to a handsome chapel presented by the same munificent Governor, are surely indications that the charge made by your Commissioner of the Radcliffe Infirmary becoming an obsolete institution is, on the very face of it, incorrect and unfounded. We will add a few words as to our surgical record of last year. Your Commissioner might have seen in the report, if he had taken the trouble to look at it, that we had twenty-one major amputations and excisions of joints (all of these recovered), five herviotomy (all recovered), six abdominal sections (two deaths), one gastrostomy (recovered), one nephrolithotomy (recovered), eight excisions of breast (one death), five excisions of thyroid (all recovered), one excision of lower jaw, one removal of tongue, besides a large number of minor operations. Anæsthetics were administered 447 times in the course of the year without an accident.

In common fairness to the Radcliffe Infirmary, and in answer to the very serious charges your commissoner has unjustly made against that institution we request you to publish this letter in your next issue.

Walter Tyrrell Brooks, M.B. Lond., M.A. Oxon.
William Collier, M.A., M.D. Cantab.
Alfred Winkfield, F.R.C.S. Eng.
Horatio P. Symonds, F.R.C.S. Edin.
W. Lewis Morgan, M.A. Oxon, M.R.C.S., L.R.C.P.

Oxford, April 14, 1891.

Our Commissioner writes: Those who compare the above letter of the medical staff with our report will see that our main criticisms are left practically untouched. These were directed against (1) the condition of the basement in the oldest part of the building, and the fittings and appliances which it contains; (2) the general aspect which it presents to the visitor on entering; (3) the absence of much real interest in the institution on the part of the inhabitants of Oxford; (4) the condition of the walls, baths, and other appliances, and especially the state of the old Parian cement in the chief surgical ward; (5) the unworthy appearance of the staircase and the entrance hall of the Jubilee wing, owing to their not having been painted; (6) certain inadequate fittings which it contains; (7) the general absence of regard for appearances in many portions of the institution; and (8) the fact that the accommodation provided for the nurses is altogether behind the times, especially as regards the cubicles and bathing arrangements. It is no answer to these criticisms to point out that certain of the outlying blocks contain some modern fittings and Rufford's baths, or to commend the excellent system of drainage which is not in question. Nor is it an answer to abuse your Commissioner, because he spoke to the nurses about their savings. The question is, "Are the above eight statements true in substance and in fact, or are they not?" We maintain that they are, and that the same conclusions will be formed by any visitor who judges the Radcliffe Infirmary from the highest standpoint of efficiency that is known to hospital administrators, and not from the aspect of thirty years ago. The main portion of the Staff's lettter relates to the Jubilee Wing and outlying blocks, which we were careful to state might "be left as they are, provided an adequate regard is shown for internal appearances in the way of re-painting and renewal," on which points your correspondents say nothing. The Staff further ignore altogether the condition of the nurses' quarters, and the necessity which exists for a properly constructed nurses' home. We have further to point out that the Staff are in error in saying that only one Sister is praised, seeing that the report speaks of all the sisters and nurses in terms of praise, and not of one only, for all are declared to be "very good." As to the statement that erysipelas and septicæmia are *almost* unknown, we have to point out that the pressure upon the beds is far less at the Oxford Infirmary than in an ordinary clinical hospital, a fact which must have an important bearing upon the results of treatment. Further, we venture to think that if the walls of the wards had been properly attended to erysipelas and septicæmia would not only have been "almost," but entirely unknown in the Radcliffe Infirmary. The Staff are no doubt unaware that we had full plans of every part of the building, and they fail to see that the criticism was a friendly one, written in what we conceive to be the best interests of the institution, and, therefore, in support of themselves and of their work, and not in any hostile spirit whatever. It is true that we judged the present condition of the Radcliffe Infirmary from the highest standpoint of hospital administration, and that so tested, in our judgment this institution is, in many respects, obsolete, so far as its present arrangements are concerned. If the medical staff, the governors, and the citizens of Oxford have any doubt on this point we would ask them to take the train to Birmingham, and to go over the new Workhouse Infirmary there. We shall be surprised if such a visit does not make them determined to at once follow the example of the county of Derby by taking steps to carry out all the much-needed improvements set forth at length in our report published in The Hospital of the 11th inst. Surely, Oxonians will never consent to allow their Infirmary, from any cause whatever within their power to remedy, to take a lower rank as to efficiency and completeness, than that held by a workhouse infirmary elsewhere.

